# A new island-scale tropical cyclone outlook for southwest Pacific nations and territories

**DOI:** 10.1038/s41598-020-67646-7

**Published:** 2020-07-21

**Authors:** Andrew D. Magee, Andrew M. Lorrey, Anthony S. Kiem, Kim Colyvas

**Affiliations:** 1 Centre for Water, Climate and Land (CWCL), University of Newcastle, Newcastle, Australia; 2 Climate, Atmosphere and Hazards Centre, National Institute for Water and Atmospheric Research (NIWA) LTD, Auckland, New Zealand; 3 School of Mathematical and Physical Sciences, University of Newcastle, Newcastle, Australia

**Keywords:** Climate sciences, Atmospheric science, Natural hazards

## Abstract

The southwest Pacific (SWP) region is vulnerable to tropical cyclone (TC) related impacts which adversely affect people, infrastructure and economies across several nations and territories. Skilful TC outlooks are needed for this region, but the erratic nature of SWP TCs and the complex ocean–atmosphere interactions that influence TC behaviour on seasonal timescales presents significant challenges. Here, we present a new TC outlook tool for the SWP using multivariate Poisson regression with indices of multiple climate modes. This approach provides skilful, island-scale TC count outlooks from July (four months ahead of the official TC season start in November). Monthly island-scale TC frequency outlooks are generated between July and December, enabling continuous refinement of predicted TC counts before and during a TC season. Use of this approach in conjunction with other seasonal climate guidance (including dynamical models) has implications for preparations ahead of severe weather events, resilience and risk reduction.

## Introduction

The southwest Pacific (SWP; 0°–35°S, 135°E–120°W) is a vast region, home to a number of diverse island nations and territories that are vulnerable to the impact of natural disasters. Tropical cyclones (TCs) account for 76% of disasters within the SWP region^1^, which bring extreme winds, intense storm surge, coastal inundation, and prolonged and intense rainfall that induces landslides and flooding^2–4^. The relative isolation of SWP islands, their high shoreline to land area ratio^5^ and the low relief of coral atolls and fringes of volcanically composed islands^6^ that are occupied by people further amplifies physical impacts from TCs. In addition, slow economic growth^6^, fragile infrastructure^7^, and a dependence on subsistence farming^8–10^ as a single primary industry has resulted in limited adaptive capacity and slower recovery times from TC impacts^11^. On ex-tropical transition, TCs have also had significant impacts on Australia^12^ and New Zealand^13^. Several severe TCs have wreaked havoc in the SWP in recent decades, devastating communities, lives and economies in the region^14–16^.

Provision of accurate and timely seasonal TC outlooks are essential for informed decision making by Pacific Island National Meteorological Services (PINMS), disaster managers and community stakeholders. For the SWP region, three seasonal TC outlooks are currently operational: (1) one produced by the National Institute for Water and Atmospheric Research (NIWA)^17^ that is distributed through the Island Climate Update^18^, (2) one from the Australian Bureau of Meteorology (BOM)^19^ and one produced by (3) the Fiji Meteorological Service (FMS)^20^. The BOM provides outlooks for two regions in the SWP, the western region (142.5°E–165°E) and the eastern region (165°E–120°W). Both NIWA and the FMS provide seasonal TC outlooks covering exclusive economic zone (EEZ) scales for SWP islands. Their products are based on selecting historical (analogue) TC seasons with similar oceanic and/or atmospheric conditions leading up to and what is expected for the season ahead. TCs within these analogue approaches are composited to infer possible conditions for the upcoming TC season and include overall TC counts, interactions of TCs with individual countries and a projection of severe TCs for the season. Each of these organisations employs a different method to derive their TC outlooks, and each considers different indices to capture ocean–atmosphere variability associated with El Niño-Southern Oscillation (ENSO).

Although ENSO is the leading mode of climate variability across the tropical Pacific^21^, it has exceptionally diverse characteristics and flavours (e.g. ENSO Modoki^22,23^) and can manifest in many different ways, both spatially and temporally^24^. Coupled ocean-atmospheric processes associated with ENSO influence changes in the location of favourable thermodynamic conditions that support tropical cyclogenesis^25,26^ , in particular guiding the South Pacific Convergence Zone^27,28^ TC incubation area systematically towards the northeast (southwest) during El Niño (La Niña) events^23,29,30^. Temporally, El Niño (La Niña) events also result in more (less) TCs during the SWP TC season, and El Niño conditions are also found to delay the onset of the following SWP TC season^30^. The diversity of ENSO means that the most suitable ENSO index/indices to underpin SWP TC outlooks may vary according to location and time of year. While numerous ENSO indices exist^31–36^, there is no consensus on which one best captures the ENSO phenomenon^37^.

Of importance, ENSO is not the only climate mode to influence SWP TC behaviour. Indian Ocean SST variability^38^ is associated with driving spatio-temporal changes in the characteristics of Australian^39,40^ and SWP TCs^41^, in conjunction with, and independent of, ENSO^42^. The co-occurrence of El Niño (La Niña) and warm (cool) SSTs in both the IOD E and IOD W regions^38^ of the tropical Indian Oceans results in significantly greater modulations of TC activity towards the northeast (southwest), compared to modulations observed through analysis of ENSO alone. The interplay between ENSO and Indian Ocean SST variability has been shown to result in substantially different risk profiles for SWP nations and territories^42^. Also, a synergy between the Southern Annular Mode (SAM) and ENSO shows an increased number of TCs undergo ex-tropical transition reaching further south during positive SAM and La Niña conditions, which is important for New Zealand^43^. Considering multivariate prediction schemes have the potential to produce more robust forecasts^44,45^, a combination of climate influences (ENSO, Indian Ocean SST variability and SAM) is highly relevant, but yet to be formally tested.

We demonstrate an approach for deriving skilful, statistically-driven island-scale TC outlooks for the SWP that incorporates ENSO with other modes of variability. Several climate indices representing inter-annual Indo-Pacific climate variability were harnessed with automated variable selection techniques to determine the most appropriate combination of model predictors for island-scale models. We also recognise the potential of additional lead time for island-scale TC outlooks is significant for PINMS, as it can enable improved decision-making for preparedness measures that can reduce TC-related risks (e.g. loss of life and infrastructure). As such, we also evaluated how changing lead times influences model skill using this approach, and compared model skill for outlooks derived in October (similar to the release timing for current operational products discussed above), with outlooks generated up to four months ahead of the TC season. In-season TC outlook updates (November–January) were also tested to determine whether refinements of TC counts for the remainder of the TC season improved efficacy.

## Results

### Deriving regional, sub-regional and island-scale TC count outlooks

**Figure 1:**
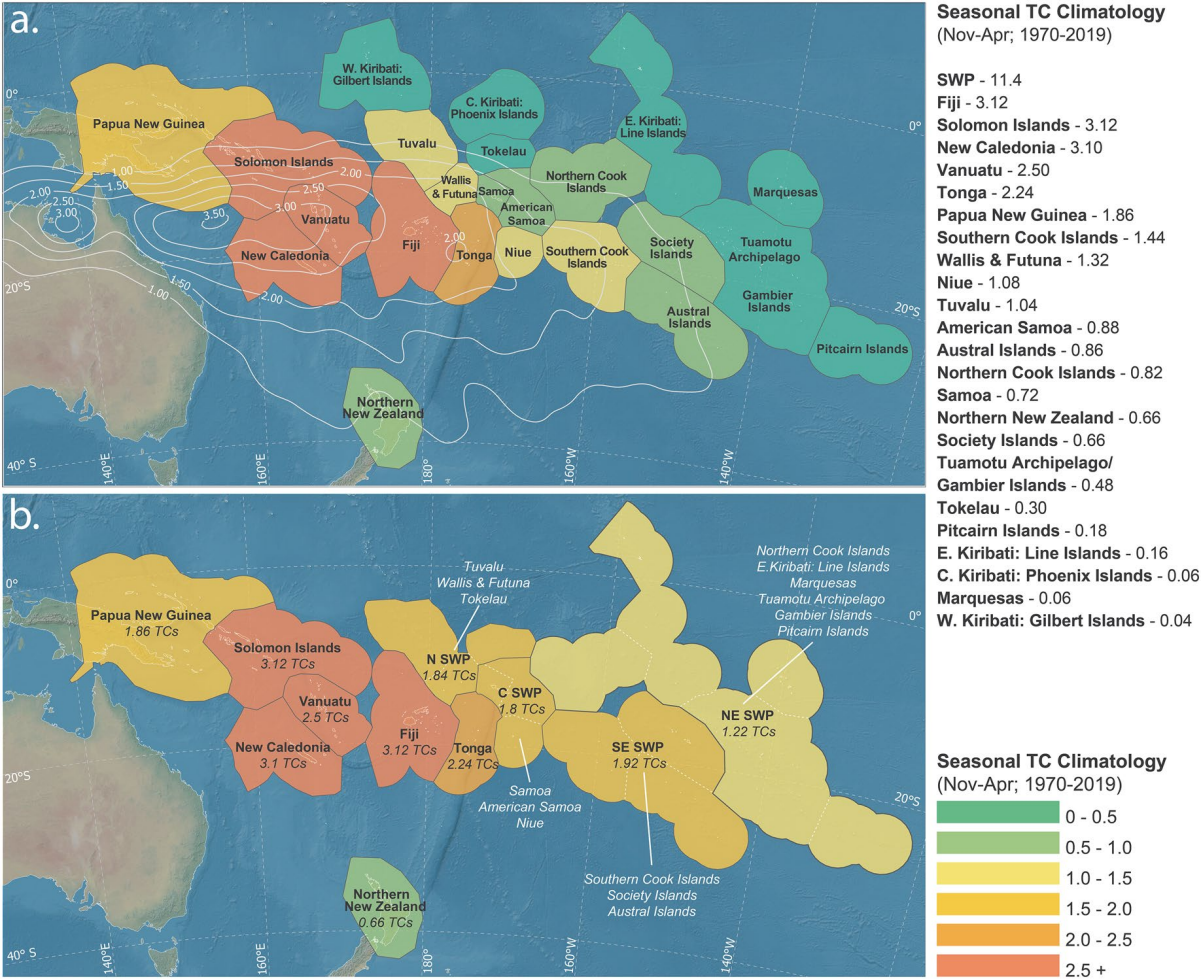
Panel a: Exclusive Economic Zones (EEZ) considered in this study with the shading seasonal TC climatology (Nov–Apr) between 1970 and 2019. Contours represent seasonal (Nov–Apr) TC track density between 1970 and 2019 (0.5 TCs/season intervals). Panel b: Location of 12 regional, sub-regional and island scale models (including entire SWP region: 0°–35°S, 135°E–120­W). Where individual EEZ climatology was < 1.5 TCs per season, surrounding EEZs were merged to form the following sub-regions: Northern SWP (N SWP; Tuvalu, Wallis & Futuna and Tokelau), Central SWP (C SWP; Samoa, American Samoa and Niue), Northeast SWP (NE SWP; Northern Cook Islands, E.Kiribati: Line Islands, Marquesas, Tuamotu Archipelago, Gambier Islands and Pitcairn Islands), and Southeast SWP (SE SWP; Southern Cook Islands, Society Islands and Austral Islands). Island-scale models were derived for the following: Papua New Guinea, Solomon Islands, Vanuatu, New Caledonia, Fiji, Tonga and Northern New Zealand. Models for W.Kiribati: Gilbert Islands and C.Kiribati: Phoenix Islands have not been derived or included as part of a larger sub-region as these locations have a low seasonal TC climatology (≤ 0.06 TCs) and are at minimal risk of TC activity. Figure created using a basemap from Natural Earth (www.naturalearthdata.com) and EEZ boundaries from^46^.

In total, 12 sub-regional and island-scale outlooks are derived for the SWP region, including a regional SWP model (see Fig. 1). The western portion of the SWP is particularly active (Fig. 1a), which supported our investigation into individual island-scale models for Fiji, Solomon Islands, New Caledonia, Vanuatu, Papua New Guinea and Tonga (November–April seasonal TC climatologies with > 1.5 TCs; see Data and Model Development section). As the eastern SWP is comparatively less active than the western SWP, EEZs in that region have been grouped together to increase the number of TC counts for sub-regional models. Our groupings have resulted in four sub-regional outlook areas: N SWP, C SWP, NE SWP and SE SWP (see Fig. 1b).

In this study, we evaluated the performance of 10 predictor models to produce TC outlooks, each of which pairs a unique ENSO index with the Marshall SAM index^47^, Indian Ocean Dipole East Box (IOD E), Indian Ocean Dipole West Box (IOD W) and the Dipole Mode Index (DMI)^38^ (see Data and Model Development and Figure S1 (supplementary) for a time series of each index used in this analysis). For predictor models 1–10 (see Table 1), an automated model selection algorithm is used to select the optimum combination of predictors (indices and lagged periods), using a generalised linear model with a Poisson distribution and log link function to model the predicted mean TCs per season for each location. Upon initiating each predictor model using the methodology as outlined in the Data and Model Development section, the model that generates the highest skill score (SS) is selected for further analysis.

**Table 1:** Ten covariate models used in analysis.

Model	ENSO index	Other indices
1	NINO1 + 2	Southern Annular Mode (SAM) IOD E IOD W Dipole Mode Index (DMI)
2	NINO3	
3	NINO3.4	
4	NINO4	
5	Southern Oscillation Index (SOI)	
6	Coupled ENSO Index (CEI)	
7	Oceanic Nino Index (ONI)	
8	Trans Nino Index (TNI)	
9	ENSO Modoki Index (EMI)	
10	ENSO Longitude Index (ELI)	

Monthly averaged lags (lags 1–6) are generated for each outlook model initiation period. Individual ENSO indices are combined with other indices including SAM, IOD E, IOD W and DMI. See the Data and Model Development Section and Fig. 6 for more details on the indices considered in this analysis.

**Figure 2:**
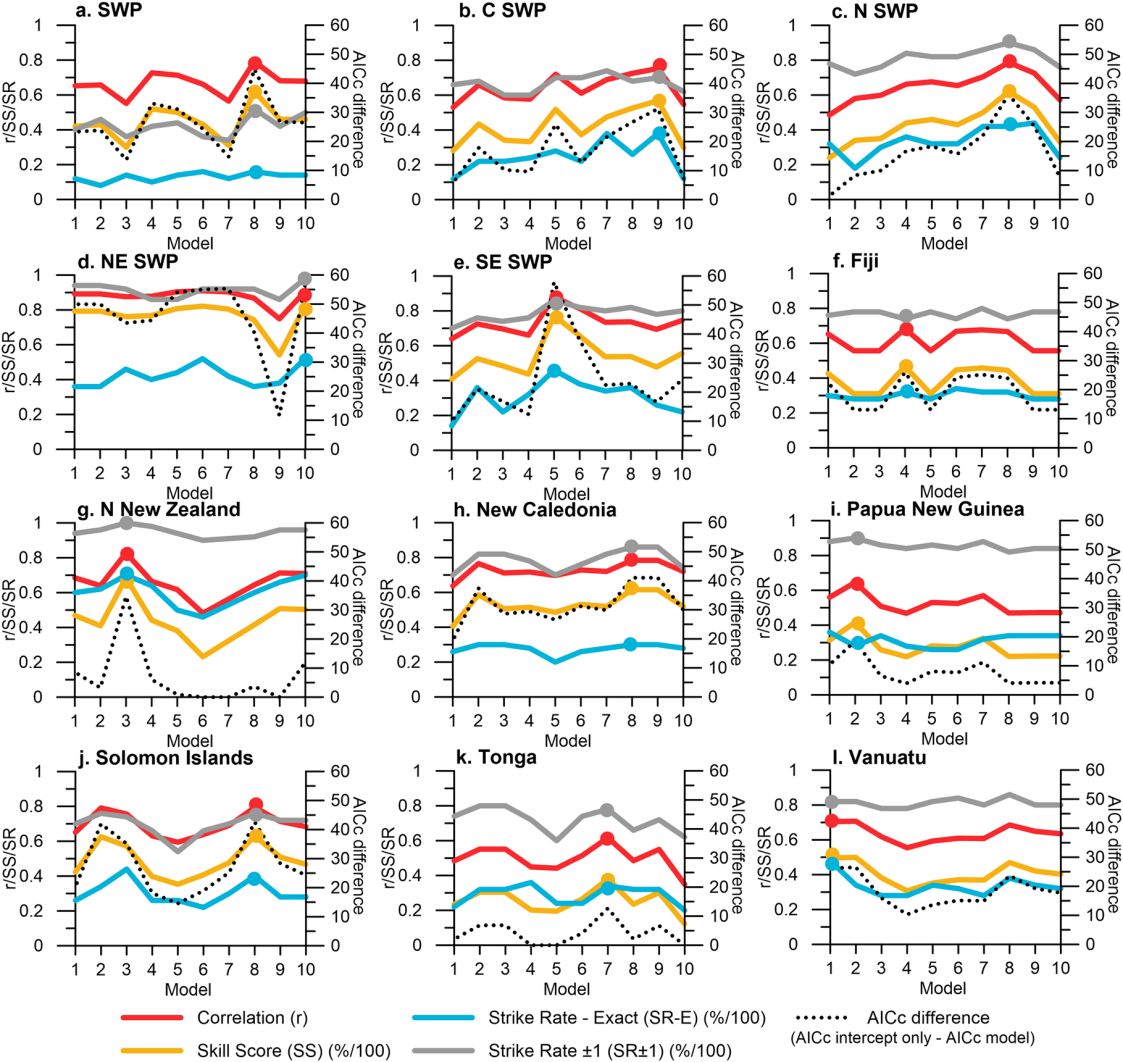
Evaluation of predictor model skill for outlooks initialised in October for the November–April TC season (see Table 1 for predictor model summary). Dots indicate models with superior model performance based on highest SS.

For TC outlooks initiated in October, the best performing models demonstrate statistically significant skill in estimating TC counts (Fig. 2), with the robustness of each model tested and cross-validated through a four-stage model calibration process (Table 2). For outlooks initiated in October, all ENSO indices (except for the Coupled ENSO Index (CEI)) were selected using the automated model selection algorithm. Of all models, the Trans Nino Index (TNI) was identified in four target areas as the most effective ENSO indicator for October initiated outlooks (SWP, N SWP, New Caledonia and the Solomon Islands), when combined with SAM, IOD E, IOD W and DMI. The methodology used to derive the TNI (the difference in normalised SST anomalies between NINO1 + 2 and NINO4)^48^, quantifies the gradient in SST anomalies between the central and eastern equatorial Pacific. Its performance in this analysis may be driven by its ability to quantify some diversity associated with ENSO events, particularly central Pacific (Modoki) ENSO events. For the Solomon Islands (3.12 TCs per season), model correlations of up to r = 0.79 (*p* =  < 0.0001, n = 50) are observed, with exact strike rates (SR-E; where the outlook, rounded to the nearest count, exactly matches the observation) of 40% (20 in 50 TC seasons) and SR ± 1 (where the outlook matches the observation ± 1 TC) of 76% (38 of 50 TC seasons). Sub-regional models also perform well and demonstrate skill in predicting TCs in regions with fewer TC events. For example, the NE SWP region (1.22 TCs per season on average) has model correlations of up to r = 0.91 (*p* =  < 0.0001, n = 50), SR-E of 52% (26 in 50 TC seasons) and SR ± 1 of 98% (49 in 50 TC seasons). This provides meteorological services in the Cook Islands, French Polynesia and Kiribati with enhanced, location-specific outlooks.

**Table 2:** Summary statistics for outlook models initiated in October for the SWP TC season (November–April).

		Sub-regional models				Island-scale models						
	SWP	C SWP	N SWP	NE SWP	SE SWP	Fiji	N New Zealand	New Caledonia	Papua New Guinea	Solomon Islands	Tonga	Vanuatu
Model number	8	9	8	10	5	4	3	8	2	8	7	1
Correlation (r)	0.79*	0.75*	0.79*	0.91*	0.88*	0.68*	0.83*	0.78*	0.65*	0.79*	0.62*	0.70*
Coefficient of determination (r^2^)	0.63	0.57	0.62	0.82	0.77	0.47	0.69	0.62	0.42	0.63	0.8	0.50
RMSE	2.30	1.28	0.97	0.73	0.89	1.17	0.50	1.09	0.99	1.24	1.27	1.10
Skill score (SS) (%)	62.7	56.7	61.9	82.1	77.25	46.7	68.4	61.08	41.67	63.05	38.03	49.63
Strike rate-exact (SR-E) (%)	16	38	42	52	46	32	70	30	28	40	34	48
Strike rate-± 1 (SR ± 1) (%)	52	70	90	98	86	74	100	86	90	76	78	82
AICc	229.37 274.76	171.24 202.54	143.26 179.46	130.86 187.42	140.35 198.25	162.07 188.07	78.48 113.19	155.38 196.41	145.27 163.80	167.77 210.32	169.90 182.58	156.40 182.62
AICc difference (intercept only-model)	45.39	31.30	36.20	56.56	57.90	26.00	34.71	41.03	18.53	42.55	12.68	26.22
Calibrate 1 1970–1994	0.84* 2.03	0.89* 0.97	0.85* 0.82	0.94* 0.70	0.92* 0.81	0.74* 1.17	1.00* 0.00	0.76* 1.17	0.67* 1.14	0.85* 11.05	0.65* 1.41	0.67* 1.24
Validate 1 1995–2019	0.56* 2.96	0.20 1.75	0.58* 1.29	0.64* 1.58	0.41^ 1.78	0.31 1.25	0.08 0.97	0.71* 1.08	0.38^ 0.96	0.29 1.68	0.35 1.28	0.64* 0.93
Calibrate 2 1995–2019	0.96* 0.99	0.91* 0.74	0.91* 0.65	0.99* 0.29	0.97* 0.48	0.70* 0.94	1.00* 0.00	0.83* 0.86	0.74* 0.70	0.89* 0.80	0.76* 0.89	0.84* 0.67
Validate 2 1970–1994	0.45^ 3.37	0.53* 1.83	0.33 1.50	0.34 1.96	0.14 2.05	0.49* 1.51	0.08 0.87	0.59* 1.45	0.18 1.50	0.31 1.88	0.48^ 1.64	0.25 1.62
Calibrate 3 1982–2006	0.86* 1.71	0.86* 1.01	0.87* 0.77	0.95* 0.38	0.99* 0.23	0.79* 0.90	1.00* 0.00	0.82* 1.14	0.84* 0.87	0.83* 1.28	0.82* 1.01	0.77* 0.92
Validate 3 1970–1981 and 2007–2019	0.45^ 3.80	0.29 1.87	0.47^ 1.46	0.85* 1.40	0.39^ 2.14	0.40^ 1.63	0.11 0.88	0.15 1.55	0.39^ 0.90	0.38^ 1.70	0.37 1.34	0.48^ 1.49
Calibrate 4 1970–1981 and 2007–2019	0.85* 2.26	0.90* 0.86	0.88* 0.80	0.90* 1.14	0.95* 0.72	0.84* 0.95	1.00* 0.00	0.89* 0.70	0.53* 0.83	0.90* 0.79	0.81* 0.84	0.71* 1.19
Validate 4 1982–2006	0.25 3.28	0.24 1.90	0.48^ 1.36	0.63* 0.91	0.32 1.53	0.49* 1.28	0.27 0.90	0.42^ 1.82	0.43^ 1.43	0.53* 1.93	0.18 1.71	0.42^ 1.30

Pearsons correlation coefficient (r), coefficient of determination (r^2^), root mean square error (RMSE), skill score (%), strike rate-exact (SR-E) (%), strike rate ± 1 (SR ± 1) (%) and finite corrected AIC (AIC_C_) summarise the performance of the derived models. For AICc, model performance (top value) is compared with the intercept only model (bottom value). Four stage twofold model validation statistics are also summarised. Correlation between IBTrACS (observed) and predicted TCs (top value) and RMSE (bottom value) summarise model performance. ^Correlation significant at 95% level. *Correlation significant at 99% level.

**Figure 3:**
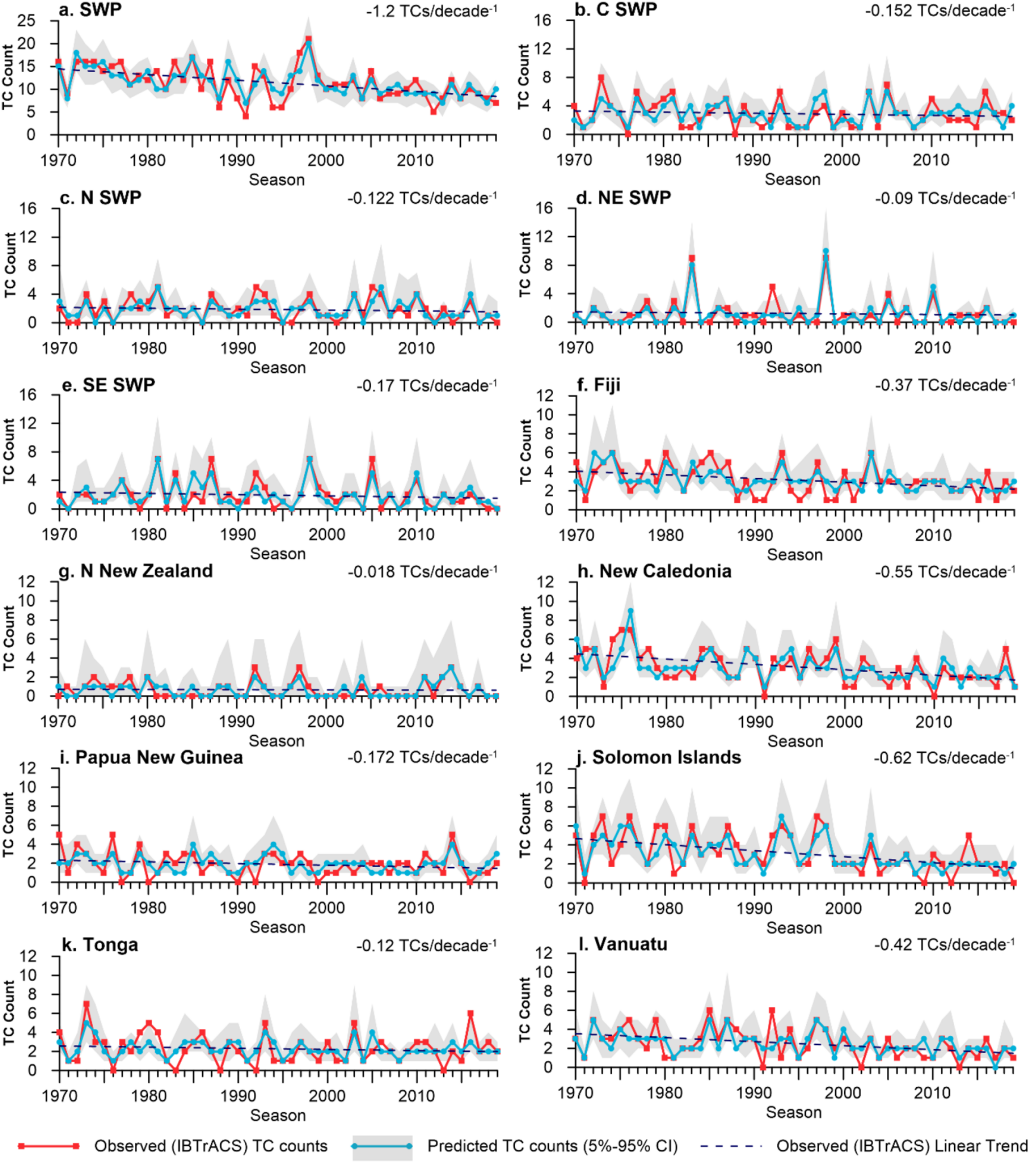
Comparison of observed (IBTrACS) TC counts and predicted TC counts for outlooks initialised in October for the following November–April TC season. 5–95% confidence intervals (CI) for predicted TC counts are shown in grey. Dashed line represents linear trend of observed (IBTrACS) TC counts.

**Figure 4:**
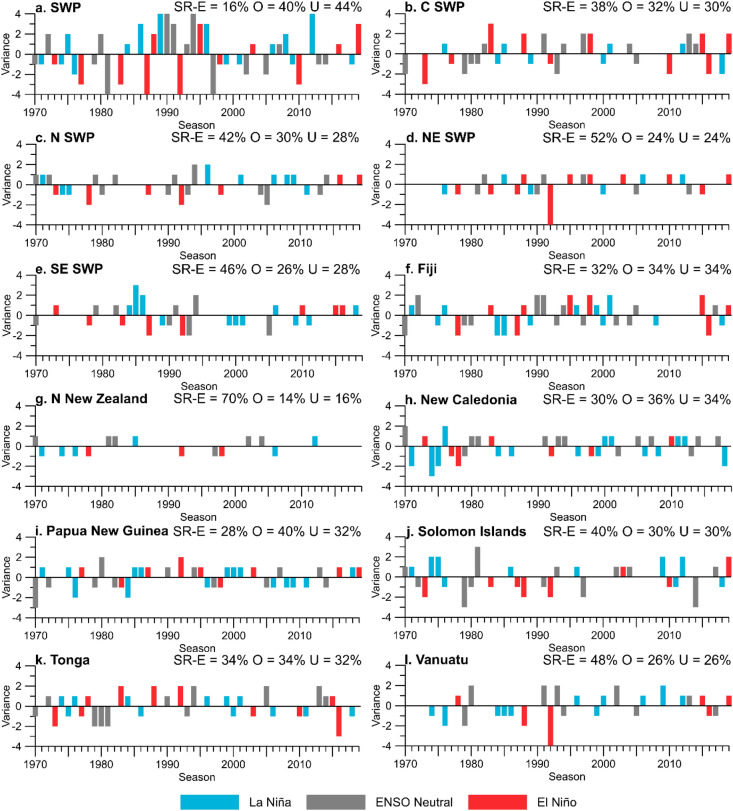
Model overestimate (O) and underestimate (U) time series for TC models initiated in October according to El Niño, La Niña and Neutral Phases (NINO3.4 Nov–Apr anomalies (1981–2010 climatology); >  + 0.5° = El Niño, < 0.5 °C = La Niña, ENSO neutral between + 0.5 °C and − 0.5 °C. O (U) indicates model has overestimated (underestimated) TC counts compared to IBTrACS observations. Exact strike rate (SR-E) is the % of time where predicted TCs and observed TCs match between 1970 and 2019. Statistics (%) for SR-E, O and U are also summarised on each panel.

Across all regional, sub-regional and island-scale models, TC count outlooks using Poisson regression are able to replicate the temporal variability and trends of observed TC counts (Fig. 3). The results capture particularly active (1998) and inactive (1991) TC seasons, as well as the decrease of TC numbers across all model target areas within the larger SWP that experienced a decrease of up to 1.2 TCs/decade^−1^ between 1970 and 2019. Although models are trained on the entire observational time series, fourfold validation was chosen intentionally to evaluate how the prediction performed when trained on one half and validated on the remaining half of the 50-year time series. For New Caledonia, calibrating the model on the first (second) half and validating on the second (first) produces statistically significant correlations of r = 0.76 (r = 0.83) and r = 0.71 (r = 0.59) respectively (*p* =  < 0.0001, n = 50 for all correlation values). The nature of this cross-validation method means it is particularly sensitive to linear trends, resulting in some non-significant validation correlations. This would not necessarily be the case for a leave-one-out cross validation (LOOCV), where the model would typically be calibrated on a much longer time period (typically n-1). Analysis of model undercount and overcount (Fig. 4), does not suggest a bias towards consistently underestimating or overestimating TC counts in a given region, nor does it reveal any bias towards a particular phase of ENSO.

### Testing model performance for monthly pre-season and in-season TC outlook updates

**Figure 5:**
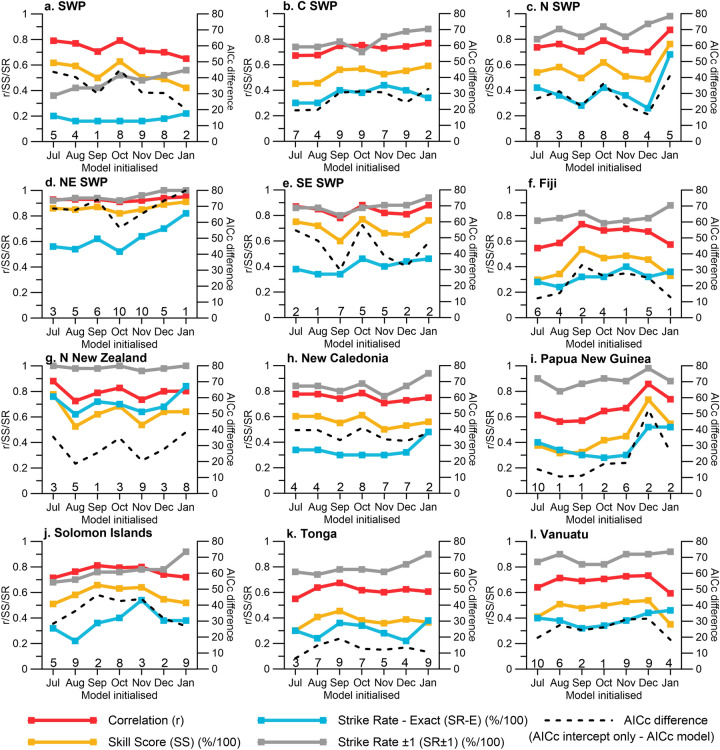
Model performance according to month of model initialisation. Models initialised in July–October predict the entire SWP TC season (November–April). In-season outlooks initialised in November, December and January, predict TCs for the remaining TC season; December–April, January–April and February–April, respectively. Numbers above x-axis indicate chosen predictor model due to superior model performance (see Table 1 for predictor model summary). See Tables S1 and S2 (supplementary material) for more information regarding model performance according to month of model initialisation.

Increasing TC outlook lead time by up to an additional three months (initialisation between July–September instead of October) can produce skilful and useful estimates of forthcoming TC activity (Fig. 5). For five out of twelve outlooks, pre-season outlook models achieved better SR-E performance when they were initiated in July versus October, e.g. SWP (24% versus 16%), NE SWP (56% versus 52%), Northern New Zealand (76% versus 70%) and Papua New Guinea (40% versus 28%). For Northern New Zealand, TC outlooks initiated in July also see higher correlation and skill score (SS) values (r = 0.88 and 78%), compared to outlooks initiated in October (r = 0.83 and 68%) (*p* =  < 0.0001, n = 50 for both correlation values). While other regions do not see improvements in model performance with increased lead time when seasonal outlooks initiated in July are compared with those initiated in October, the models initiated in July still perform well.

Model performance of in-season TC outlooks (November–January) is also tested. In-season TC guidance updates offers an opportunity to refine outlooks for the late season, which is important given the second half of the SWP TC season is typically more active than the first half^49^. In-season models perform well, with notable improvements in SR-E initiated in October versus January; e.g. 42% versus 68% for N SWP, 52% versus 82% for NE SWP, and 28% versus 52% for Papua New Guinea outlooks. On average and across all regional, sub-regional and island-scale models, SR-E (SR ± 1) increases from 39% (81%) in October to 50% (90%) for models initialised in January.

Analysis of the proportionality of covariates for regional, sub-regional and island-scale models (Table 3) shows that indices representing Indian Ocean SST variability (particularly IOD E and IOD W) dominate predictor model covariate selection, accounting for between 36% (Papua New Guinea) and 54.2% (Vanuatu) of predictors. For ENSO, both NINO3 and EMI were identified as preferred models for four locations, while the TNI was identified as the most common ENSO predictor for two locations (SWP and N SWP). Concomitant with the location of central Pacific (ENSO Modoki) events in the Pacific, the EMI is identified as the most favourable ENSO index (14.5%) for C SWP TCs. Table 3 and Fig. 5 show that all ENSO indices are selected as a superior model at least once, highlighting the importance of including multiple ENSO indices to represent the complex ocean–atmosphere interactions associated with the phenomenon and location-specific outcomes^42,50^. SAM accounts for 11.1% (New Caledonia) to 30.3% (C SWP) of covariates, confirming it is an important climate mode to consider in order to improve the predictive skill of TC outlook models.

**Table 3:** Proportion (%) of model covariates for outlook model runs from July–January.

Index		Subregional models				Island-scale models						
	SWP	C SWP	N SWP	NE SWP	SE SWP	Fiji	N New Zealand	New Caledonia	Papua New Guinea	Solomon Islands	Tonga	Vanuatu
NINO1 + 2	3.3	0	0	6.3	2.3	7.4	3.8	0	8.0	0	0	5.1
NINO3	0	3.9	0	0	**11.5**	5.6	0	**11.1**	**14.0**	**7.7**	0	5.1
NINO3.4	0	0	2.8	3.8	0	0	**10.3**	0	0	4.6	3.5	0
NINO4	6.7	1.3	5.6	0	0	**11.1**	0	8.9	0	0	3.5	3.4
SOI	0	0	5.6	3.8	9.2	3.7	3.8	0	0	4.6	3.5	0
CEI	0	0	0	6.3	0	5.6	0	0	8.0	0	0	6.8
ONI	0	9.2	0	0	3.4	0	0	8.9	0	0	7.0	0
TNI	**13.3**	0	**19.4**	0	0	0	5.1	8.9	0	6.2	0	0
EMI	3.3	**14.5**	0	0	0	0	3.8	0	0	**7.7**	**10.5**	**13.6**
ELI	5.0	0	0	**10.1**	0	0	0	0	10.0	0	0	1.7
SAM	23.3	30.3	18.1	24.1	26.4	27.8	26.9	11.1	24.0	20.0	28.1	10.2
IOD E	21.7	17.1	18.1	24.1	27.6	9.3	23.1	22.2	16.0	24.6	19.3	27.1
IOD W	23.3	22.4	26.4	21.5	18.4	29.6	23.1	26.7	20.0	24.6	24.6	27.1
DMI	0	1.3	4.2	0	1.1	0	0	2.2	0	0	0	0

Bold values indicates ENSO index with the largest proportional contribution per location.

## Discussion

We have derived and tested Poisson regression that uses indices representing multiple modes of climate variability for bespoke island-scale and sub-regional scale TC guidance. This approach can provide up to three months additional lead time compared to current operational regional TC seasonal outlooks. We tested model performance across a number of initialisation periods and found that model skill was sufficient to enable TC count outlooks prior to (from as early as July) and during the early (November–January) SWP TC season (which is designated by the PINMS as including November–April). In-season monthly TC count outlooks generated using the method presented here indicates that the later the outlooks are generated, the more accurate they are.

Compared to other studies that explore various methodologies to derive TC forecasts for the SWP, including simple linear regression approaches^51^, Bayesian regression^52^, Poisson regression^53,54^, and machine learning algorithms^45^, the method presented in this analysis is unique in a number of ways. First, for each predictor model, the automated covariate selection algorithm enabled the optimum combination of five Indo-Pacific climate indices, each of which has six-monthly lags (30 covariates in total). Second, given ENSO is the dominant mode of variability in the Pacific^55^, and the well-established ENSO-TC frequency relationship^23,29,30^, 10 unique predictor models (each of which contained a unique ENSO index) are tested for each regional, sub-regional and island-scale location. Superior models were selected using the highest SS (see Data and Model Development). Inclusion of an extensive range of ENSO indices circumvents issues surrounding subjectivity in choosing an ENSO index, which has the potential to limit a model’s prediction potential. Third, the results shown in Table 3 show that the average proportional contribution of indices contained across all regional, sub-regional and island-scale models account for 45.6% (Indian Ocean SST variability; IOD E, IOD W and DMI), 31.9% (ENSO) and 22.5% (SAM). For every sub-regional/island-scale model and model initialisation period, every ENSO index was used at least once. While not every mode of tropical and extratropical variability could be included in this analysis, considering ENSO, SAM and Indian Ocean SST variability and the interactions between them, has proven to add skill across all regional, sub-regional and island-scale models and initialisation periods.

The benefits of generating independent, location-specific TC outlook models using Poisson regression are wide-reaching, and not confined to the SWP. They have potential to provide skilful island-scale TC count estimates for each season at a variety of lead times, and this approach can potentially be adapted to other ocean basins (as well as other time-transgressive geospatial datasets) where multiple driving factors for storm activity come into play. For the SWP, this new approach provides a complementary perspective to regional outlooks from official forecasting agencies that only consider how regional TC activity may impact island nations and territories. Island-scale and sub-regional scale outlooks based on Poisson regression outlooks for TCs also have the potential to improve testing of storm count strike rates because calibration and verification for this method is undertaken over a finer spatial scale than what is used for present regional TC outlooks. From this perspective, our new approach adds an additional layer of validated guidance relative to extant statistical and dynamical TC outlook products. In doing so, this addition strengthens the prospect for SWP ensemble-based guidance for TC activity.

The methodology outlined in this paper can also be applied, updated and retrained to incorporate storm counts from the most recent TC seasons. As such, we expect future improvements for the skill and reduction of uncertainty for island-scale TC outlooks using this approach. In addition, the ability to easily re-run the models every month to include the most recent ocean–atmosphere conditions (model covariates), means TC guidance can be updated on a monthly basis between July and January to cover the SWP TC season that lies ahead. This is expected to help bridge current sub-seasonal and seasonal climate guidance that indicates where storm activity may be elevated or reduced, which can change quickly depending on intra-seasonal ENSO developments. This guidance will be updated and freely available on the Tropical Cyclone Outlook for the Southwest Pacific (TCO-SP) website (www.tcoutlook.com), to support end-users (including meteorological and government agencies, civil defence managers, non-governmental aid organisations and the general public) who can access it in support of decision making and to promote the benefits of expanding early warning systems for weather extremes.

## Data and model development

### Tropical cyclone data

This study uses TC best-track data from the International Best-Track Archive for Climate Stewardship (IBTrACSv4)^56^ for the southwest Pacific (SWP; 0–35°S, 135°E–120°W). The SWP TC season extends from November to April (the following year). While TCs can occur outside of the SWP TC season, we only consider events that occur within the SWP TC season. Only events where sustained winds of > 34 kt (63 km/h) are included in this analysis.

Exclusive Economic Zones (EEZs) are used to delineate island group boundaries. In total, 23 EEZs exist in the SWP region (Fig. 1a). The number of TCs to pass within each EEZ is calculated on a monthly basis (Nov–Apr) between 1970 and 2019. Seasonal TC climatologies (Fig. 1a), range from 3.12 TCs per season (Fiji) to 0.06 TCs per season (C. Kiribati and Marquesas). A threshold of 1.5 TCs per season is used to determine which EEZ should have an individual outlook model, and which EEZs may suffer from insignificant skill due to small sample size. Where the seasonal TC climatology < 1.5, geographically neighbouring EEZs are grouped together (Fig. 1b). Northern New Zealand is an exception as its relative isolation does not allow it to be merged with another EEZ. Seven individual island-scale outlooks are derived and include Papua New Guinea, Solomon Islands, New Caledonia, Vanuatu, Fiji, Tonga and Northern New Zealand. Where seasonal TC climatology < 1.5, four sub-regional EEZ outlooks are derived: northern SWP (N SWP; 1.84 TCs per season), central SWP (C SWP; 1.8 TCs per season), northeastern SWP (NE SWP; 1.22 TCs per season) and southeastern SWP (SE SWP; 1.92 TCs). The split between the NE SWP and SE SWP was influenced by the average location of the SPCZ, an important component that influences regional climate^57^. A model is also derived for the entire SWP basin. The Gilbert and Phoenix Islands (Kiribati) have not been included in this analysis as these regions are at minimal risk of TC activity. In total, twelve outlooks are derived and validated in this analysis.

### Model covariates

**Figure 6:**
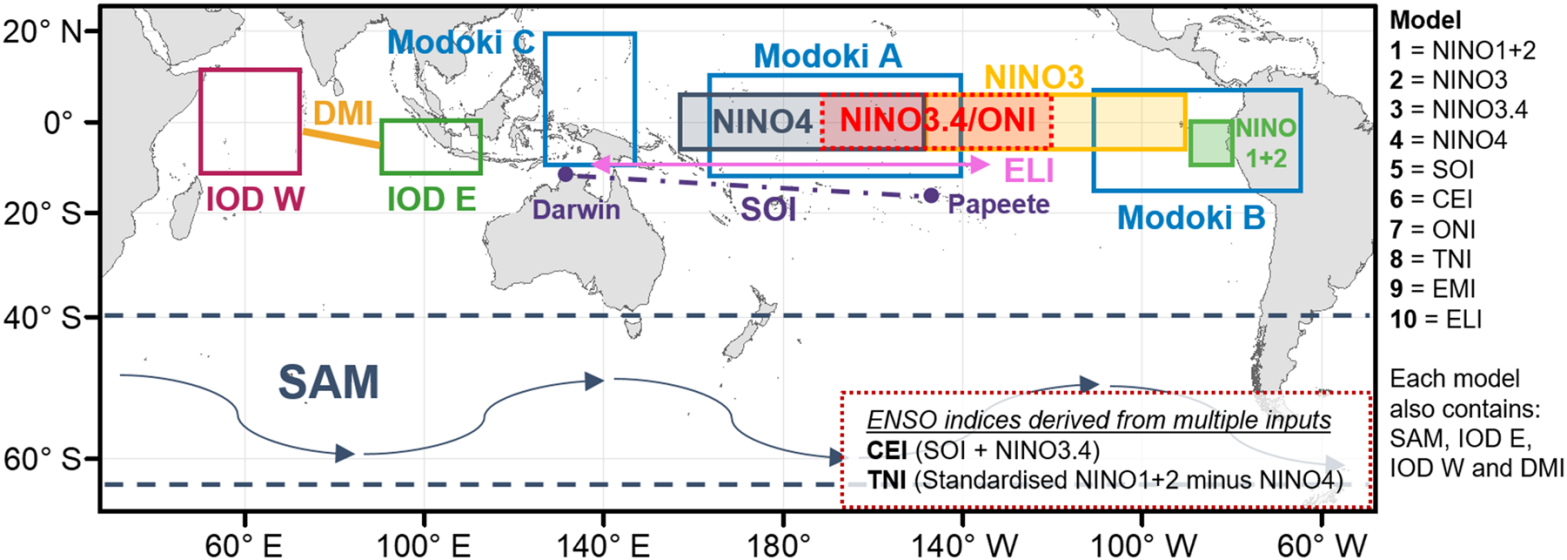
Indo-Pacific climate indices (model covariates) considered in this analysis. Indices representing El Niño-Southern Oscillation (ENSO) include: NINO1 + 2, NINO3, NINO4, NINO3.4, Southern Oscillation Index (SOI), ENSO Modoki Index (EMI), Coupled ENSO Index (CEI), Oceanic Nino Index (ONI), Trans NINO Index (TNI) and ENSO Longitude Index (ELI). Other indices considered include the Southern Annular Mode (SAM), Indian Ocean Dipole West pole (IOD W), Indian Ocean Dipole East pole (IOD E), and the Dipole Mode Index (DMI). Ten predictor model combinations used in analysis are summarised to the right of the panel and in Table 1. Monthly averaged lags (lag 1–6) are generated for each outlook initiation period. Basemap from Natural Earth (www.naturalearthdata.com).

A total of 14 monthly indices representing Indo-Pacific climate variability are used in this analysis (see Fig. 6). Only one ENSO indicator at a time is paired with indices 2–5 below, resulting in ten unique predictor models. Further details are outlined below:

1. ENSO: Ten ENSO indices are evaluated in this analysis and include (a) NINO1 + 2^48^, (b) NINO3^48^, (c) NINO3.4^58^, (d) NINO4^32^, (e) Southern Oscillation Index (SOI)^33^, (f) Coupled ENSO Index (CEI)^34^, (g) Oceanic NINO Index (ONI)^35^, (h) Trans Nino Index (TNI)^48^, (i) ENSO Modoki Index (EMI)^59^, and (j) ENSO Longitude Index (ELI)^36^. All oceanic based indices are calculated using ERSSTv5^60^. In calculating the CEI, we use a 3-month smoothed version with anomalies calculated using a 1970–2018 climatology. See Fig. 6 for a diagrammatic representation of all indices included for analysis.
2. Marshall SAM Index: a station-based index based on zonal pressure differences between 40°S and 65°S^47^.
3. IOD E: SST anomalies in the IOD E region (eastern pole of DMI; 0°–10°S, 90°E–110°E^38^.
4. IOD W: SST anomalies in the IOD W region (western pole of DMI; 10°N–10°S, 50°E–70°E^38^.
5. DMI: the difference in SST anomalies between IOD W and IOD E^38^.

### Model development: deriving regional, sub-regional and island-scale TC outlooks

Poisson regression, a special case of generalised linear modelling (GLM) is used to calculate the contribution of model covariates to predict observed TCs, *y,* during the training period between 1970 and 2019 (50 seasons in total)*.* Consistent with other studies^44,53,54,61–63^, we follow a Poisson distributional process is modelling TC counts given their discrete nature^61^. As such,

1 $$P\left({Y}_{i}=y\right)= \frac{{\mu }_{i}^{y}\mathrm{exp}(-{\mu }_{i})}{y!}, y=0, 1, 2, \dots $$

where:

2 $${\mu }_{i}=\mathrm{exp}\left({\beta }_{0}+ \sum_{j}\left({\beta }_{j}{x}_{ij}\right)\right)$$

where μ_*i*_ is the expected number of TC counts with covariate values ${x}_{ij}$ for the $j$ predictors on the $i$ th observation. ${\beta }_{j}$ refers to the regression coefficient for each covariate and ${\beta }_{o}$, the intercept.

Prior to variable selection, each predictor model contains six consecutive one-month values (lags). For example, models initiated in October will have values from lag 1 (September) through to lag 6 (April) and models initiated in July, lag 1 is June and lag 6 is January. For each of the 10 predictor models, there are 30 variables (5 indices with 6 monthly lags). Variable selection is used to identify the most appropriate combination of predictors for each of the ten predictor models per EEZ. Stepwise model selection is performed using the stepAIC function in the MASS R package^64^. Forward and backward elimination was used to successively include and/or remove variables using the AIC (Akaike Information Criterion^65^) as a selection criterion for choosing when the variable elimination procedure should stop. Poisson regression is then applied to these selected covariates to derive an outlook TC timeseries. Checks on the mean–variance relationship over all EEZs and on the total TC count for lag-1 serial correlation and overdispersion confirmed that Poisson regression was appropriate.

Model validation was then used to evaluate the performance and predictive skill of each model^66^. Twofold cross validation is used to evaluate the performance and predictive skill of each model^66^. Applying twofold cross validation four times, the 50-year time series is divided into two 25-year blocks and model performance is evaluated. Calibration is performed four times: on the first half (1970–1994), the second half (1995–2019), the middle half (1982–2006) and on a split between the start (1970–1981) and end (2007–2019) of the time series. Validation is subsequently performed on the remaining periods. This method of cross-validation evaluates how well the model performs when trained on only half of the 50-year time series. The selected covariates from the variable elimination procedure above were the starting point for the training phase to evaluate real-time outlooks skill, and assesses how well a model is able to replicate decreasing trends in TC counts (see Fig. 3).

The following performance measures were used to evaluate the predictive skill of the TC outlooks compared to the observations: the Pearson correlation coefficient (predicted TCs versus observed TCs), root mean square error of the prediction (RMSE), strike rate (SR) exact (SR-exact) (% of time the prediction (rounded to the nearest whole number) matches the observation) and SR ± 1 (as per exact, but where prediction is ± 1 from observation). SR ± 1 is sensitive to the mean and variance of TC counts for a given forecast region and is more likely to be high if the overall mean and variance of TC counts is low. This should be taken into consideration when using this statistic. Skill score (SS), an evaluation of model performance is also calculated^67^, where 100% represents a perfect outlook and 0% represents outlooks as accurate as the climatology. The finite sample corrected Akaike information criterion (AIC_C_) is used to estimate the quality of a model relative to another^65^.

For each island-scale TC outlook, the skill of each of the ten predictor models is evaluated before the best performing model is selected. Given this study also tests how the skill of TC outlooks change depending on when the model is initialised, lag 1 is always one month before the model initialisation month. The study completes the above methodology for each of the 10 predictor models (Table 1) and considers 7 different model initialisation months (July–January) for each of the 12 regional, sub-regional and island-scale outlooks (840 model runs in total).

## Supplementary information

Supplementary file1 (DOCX 266 kb)
