# Supplementary material for: A new island-scale tropical cyclone outlook for southwest Pacific nations and territories
Source: Sci Rep. 2020 Jul 21;10:11286. doi: 10.1038/s41598-020-67646-7 (PMC7374748; doi:10.1038/s41598-020-67646-7)
Supplement: Supplementary file 1 — Supplementary file1 (DOCX 266 kb) [file 41598_2020_67646_MOESM1_ESM.docx]

**A new island-scale tropical cyclone outlook for southwest Pacific nations and territories**

Andrew D. Magee^1*^, Andrew M. Lorrey^2^, Anthony S. Kiem^1^, Kim Colyvas^3^

^1^ Centre for Water, Climate and Land (CWCL), University of Newcastle, Australia

^2^ Climate, Atmosphere and Hazards Centre, National Institute for Water and Atmospheric Research (NIWA) LTD, New Zealand

^3^ School of Mathematical and Physical Sciences, University of Newcastle, Australia

* Corresponding author: [andrew.magee@newcastle.edu.au](mailto:andrew.magee@newcastle.edu.au)

**Supplementary Material**

**Table S1.** Summary statistics for sub-region pre-season outlook models initiated each month between July-October for the November-April TC season and in-season outlook models initiated each month between November, December and January for the remaining December-April, January-April and February-April TC season, respectively. Pearsons correlation coefficient (r), R square, Root Mean Square Error (RMSE), Skill Score (%), Strike rate – exact (%), Strike rate ±1 (%) and Finite Corrected AIC (AIC_C_) summarise the performance of the derived models. For AICc, model performance (top value) is compared with the intercept only model (bottom value). Four stage two-fold model validation statistics are also summarised. Correlation between IBTrACS (observed) and predicted TCs (top value) and RMSE (bottom value) summarise model performance. ^ correlation significant at 95% level. * correlation significant at 99% level.

|  |  | **Pre-Season** | | | | **In-Season** | | |
| --- | --- | --- | --- | --- | --- | --- | --- | --- |
|  |  | **Jul** | **Aug** | **Sep** | **Oct** | **Nov** | **Dec** | **Jan** |
| **SWP** | **Model Number** | 5 | 4 | 1 | 8 | 9 | 8 | 2 |
|  | **Correlation (r)** | 0.79 | 0.77 | 0.71 | 0.79 | 0.71 | 0.70 | 0.65 |
|  | **R-square** | 0.62 | 0.59 | 0.50 | 0.63 | 0.50 | 0.49 | 0.42 |
|  | **RMSE** | 2.33 | 2.41 | 2.67 | 2.30 | 2.44 | 2.32 | 2.03 |
|  | **Skill Score (%)** | 61.77 | 59.23 | 49.82 | 62.73 | 50.39 | 49.08 | 42.01 |
|  | **Strike Rate – exact (%)** | 20 | 16 | 16 | 16 | 16 | 18 | 22 |
|  | **Strike Rate - ±1 (%)** | 36 | 42 | 42 | 52 | 48 | 52 | 56 |
|  | **AICc** | 231.12  274.76 | 234.34  274.76 | 244.75  274.76 | 229.87  274.76 | 235.57  266.24 | 230.57  260.99 | 217.32  239.87 |
|  | **AICc difference**  **(model-intercept only)** | 43.64 | 40.42 | 30.01 | 44.90 | 30.67 | 30.42 | 22.55 |
| **C SWP** | **Model Number** | 7 | 4 | 9 | 9 | 7 | 9 | 2 |
|  | **Correlation (r)** | 0.67 | 0.67 | 0.75 | 0.75 | 0.76 | 0.74 | 0.77 |
|  | **R-square** | 0.45 | 0.46 | 0.56 | 0.57 | 0.57 | 0.55 | 0.59 |
|  | **RMSE** | 1.44 | 1.44 | 1.29 | 1.28 | 1.04 | 1.03 | 1.02 |
|  | **Skill Score (%)** | 45.11 | 45.50 | 56.00 | 56.74 | 57.33 | 55.26 | 59.00 |
|  | **Strike Rate – exact (%)** | 30 | 30 | 40 | 38 | 38 | 40 | 34 |
|  | **Strike Rate - ±1 (%)** | 74 | 74 | 78 | 70 | 84 | 86 | 88 |
|  | **AICc** | 183.14  202.54 | 182.8  202.54 | 172.04  202.54 | 171.24  202.54 | 150.25  180.91 | 149.58  173.92 | 148.15  180.91 |
|  | **AICc difference**  **(model-intercept only)** | 19.40 | 19.74 | 30.50 | 31.31 | 30.66 | 24.34 | 32.76 |
| **N SWP** | **Model Number** | 8 | 3 | 8 | 8 | 8 | 4 | 5 |
|  | **Correlation (r)** | 0.74 | 0.76 | 0.70 | 0.79 | 0.71 | 0.70 | 0.87 |
|  | **R-square** | 0.54 | 0.58 | 0.50 | 0.62 | 0.51 | 0.49 | 0.76 |
|  | **RMSE** | 1.06 | 1.02 | 1.12 | 0.97 | 1.06 | 0.98 | 0.54 |
|  | **Skill Score (%)** | 54.10 | 58.12 | 49.60 | 61.91 | 50.92 | 48.87 | 76.23 |
|  | **Strike Rate – exact (%)** | 42 | 36 | 28 | 42 | 36 | 26 | 68 |
|  | **Strike Rate - ±1 (%)** | 80 | 88 | 82 | 90 | 82 | 92 | 98 |
|  | **AICc** | 152.57  179.46 | 148.13  179.46 | 157.35  179.46 | 143.26  179.46 | 152.28  174.48 | 143.92  159.10 | 85.44  126.51 |
|  | **AICc difference**  **(model-intercept only)** | 26.89 | 31.33 | 22.11 | 36.20 | 22.19 | 15.18 | 41.07 |
| **NE SWP** | **Model Number** | 3 | 5 | 6 | 10 | 10 | 5 | 1 |
|  | **Correlation (r)** | 0.93 | 0.93 | 0.93 | 0.91 | 0.92 | 0.94 | 0.95 |
|  | **R-square** | 0.86 | 0.86 | 0.72 | 0.82 | 0.85 | 0.89 | 0.91 |
|  | **RMSE** | 0.76 | 0.77 | 0.87 | 0.86 | 0.71 | 0.53 | 0.43 |
|  | **Skill Score (%)** | 85.94 | 85.12 | 87.13 | 82.10 | 85.39 | 88.89 | 90.91 |
|  | **Strike Rate – exact (%)** | 56 | 54 | 62 | 52 | 64 | 70 | 82 |
|  | **Strike Rate - ±1 (%)** | 92 | 94 | 94 | 98 | 96 | 100 | 100 |
|  | **AICc** | 118.78  187.42 | 119.76  187.42 | 113.24  187.42 | 130.86  187.42 | 112.44  177.71 | 81.98  155.44 | 61.37  141.57 |
|  | **AICc difference**  **(model-intercept only)** | 68.64 | 67.66 | 74.18 | 56.56 | 65.27 | 73.45 | 80.20 |
| **SE SWP** | **Model Number** | 2 | 1 | 7 | 5 | 5 | 2 | 2 |
|  | **Correlation (r)** | 0.87 | 0.85 | 0.78 | 0.88 | 0.82 | 0.81 | 0.88 |
|  | **R-square** | 0.76 | 0.72 | 0.60 | 0.77 | 0.66 | 0.65 | 0.77 |
|  | **RMSE** | 0.97 | 1.04 | 1.25 | 0.94 | 1.07 | 0.99 | 0.73 |
|  | **Skill Score (%)** | 75.45 | 71.93 | 59.83 | 77.25 | 66.35 | 64.78 | 75.68 |
|  | **Strike Rate – exact (%)** | 38 | 34 | 34 | 46 | 40 | 44 | 46 |
|  | **Strike Rate - ±1 (%)** | 86 | 86 | 80 | 86 | 88 | 88 | 94 |
|  | **AICc** | 143.67  198.25 | 150.18  198.25 | 168.46  198.25 | 140.35  198.25 | 153.45  191.93 | 145.54  177.72 | 114.63  161.72 |
|  | **AICc difference**  **(model-intercept only)** | 54.58 | 48.07 | 29.78 | 57.90 | 38.48 | 32.18 | 47.08 |

**Table S2.** Summary statistics for Island-scale pre-season outlook models initiated each month between July-October for the November-April TC season and in-season outlook models initiated each month between November, December and January for the remaining December-April, January-April and February-April TC season, respectively. Pearsons correlation coefficient (r), R square, Root Mean Square Error (RMSE), Skill Score (%), Strike rate – exact (%), Strike rate ±1 (%) and Finite Corrected AIC (AIC_C_) summarise the performance of the derived models. For AICc, model performance (top value) is compared with the intercept only model (bottom value). Four stage two-fold model validation statistics are also summarised. Correlation between IBTrACS (observed) and predicted TCs (top value) and RMSE (bottom value) summarise model performance. ^ correlation significant at 95% level. * correlation significant at 99% level.

|  |  | **Pre-Season** | | | | **In-Season** | | |
| --- | --- | --- | --- | --- | --- | --- | --- | --- |
|  |  | **Jul** | **Aug** | **Sep** | **Oct** | **Nov** | **Dec** | **Jan** |
| **Fiji** | **Model Number** | 6 | 4 | 2 | 4 | 1 | 5 | 1 |
|  | **Correlation (r)** | 0.55 | 0.58 | 0.73 | 0.68 | 0.70 | 0.68 | 0.57 |
|  | **R-square** | 0.30 | 0.34 | 0.54 | 0.47 | 0.48 | 0.46 | 0.33 |
|  | **RMSE** | 1.34 | 1.30 | 1.09 | 1.17 | 1.16 | 1.19 | 1.02 |
|  | **Skill Score (%)** | 29.71 | 34.16 | 53.56 | 46.69 | 48.47 | 45.58 | 32.83 |
|  | **Strike Rate – exact (%)** | 28 | 24 | 32 | 32 | 40 | 32 | 36 |
|  | **Strike Rate - ±1 (%)** | 76 | 78 | 82 | 74 | 76 | 78 | 88 |
|  | **AICc** | 175.90  188.07 | 172.64  188.07 | 155.13  188.07 | 162.08  188.07 | 160.99  188.85 | 163.74  188.85 | 148.12  160.98 |
|  | **AICc difference**  **(model-intercept only)** | 12.18 | 15.43 | 32.94 | 26.00 | 27.86 | 25.11 | 12.86 |
| **N New Zealand** | **Model Number** | 3 | 5 | 1 | 3 | 9 | 3 | 8 |
|  | **Correlation (r)** | 0.88 | 0.75 | 0.79 | 0.83 | 0.76 | 0.80 | 0.80 |
|  | **R-square** | 0.78 | 0.57 | 0.62 | 0.69 | 0.58 | 0.64 | 0.64 |
|  | **RMSE** | 0.43 | 0.60 | 0.56 | 0.51 | 0.58 | 0.50 | 0.37 |
|  | **Skill Score (%)** | 77.58 | 56.08 | 62.14 | 68.38 | 58.15 | 64.04 | 64.13 |
|  | **Strike Rate – exact (%)** | 76 | 62 | 72 | 70 | 60 | 68 | 84 |
|  | **Strike Rate - ±1 (%)** | 100 | 98 | 98 | 100 | 98 | 98 | 100 |
|  | **AICc** | 61.27  113.19 | 94.52  113.19 | 87.70  113.19 | 78.48  113.19 | 92.67  113.19 | 78.05  105.78 | 46.21  84.68 |
|  | **AICc difference**  **(model-intercept only)** | 51.92 | 18.67 | 25.49 | 34.71 | 20.52 | 27.73 | 38.46 |
| **New Caledonia** | **Model Number** | 4 | 4 | 2 | 8 | 7 | 7 | 2 |
|  | **Correlation (r)** | 0.78 | 0.78 | 0.74 | 0.78 | 0.74 | 0.73 | 0.75 |
|  | **R-square** | 0.60 | 0.60 | 0.55 | 0.62 | 0.55 | 0.53 | 0.56 |
|  | **RMSE** | 1.11 | 1.11 | 1.18 | 1.09 | 1.13 | 1.07 | 0.79 |
|  | **Skill Score (%)** | 60.27 | 60.27 | 55.07 | 61.08 | 54.63 | 52.99 | 55.87 |
|  | **Strike Rate – exact (%)** | 34 | 34 | 30 | 30 | 22 | 32 | 48 |
|  | **Strike Rate - ±1 (%)** | 84 | 84 | 80 | 86 | 88 | 84 | 94 |
|  | **AICc** | 156.85  196.41 | 156.85  196.41 | 163.10  196.41 | 155.38  196.41 | 158.42  192.25 | 153.59  186.35 | 122.72  160.49 |
|  | **AICc difference**  **(model-intercept only)** | 39.56 | 39.56 | 33.31 | 41.03 | 33.83 | 32.76 | 37.77 |
| **Papua New Guinea** | **Model Number** | 10 | 1 | 1 | 2 | 6 | 2 | 2 |
|  | **Correlation (r)** | 0.61 | 0.56 | 0.57 | 0.65 | 0.67 | 0.86 | 0.74 |
|  | **R-square** | 0.38 | 0.32 | 0.32 | 0.42 | 0.45 | 0.74 | 0.54 |
|  | **RMSE** | 1.02 | 1.07 | 1.06 | 0.99 | 0.99 | 0.70 | 0.80 |
|  | **Skill Score (%)** | 37.58 | 31.59 | 32.31 | 41.67 | 44.76 | 73.50 | 54.46 |
|  | **Strike Rate – exact (%)** | 40 | 34 | 30 | 28 | 30 | 52 | 52 |
|  | **Strike Rate - ±1 (%)** | 90 | 80 | 86 | 90 | 88 | 98 | 88 |
|  | **AICc** | 148.66  163.80 | 153.24  163.80 | 152.65  163.80 | 145.27  163.80 | 145.86  164.98 | 110.07  162.41 | 123.87  149.668 |
|  | **AICc difference**  **(model-intercept only)** | 15.14 | 10.56 | 11.15 | 18.53 | 19.13 | 52.35 | 25.81 |
| **Solomon Islands** | **Model Number** | 5 | 9 | 2 | 8 | 3 | 2 | 9 |
|  | **Correlation (r)** | 0.71 | 0.76 | 0.81 | 0.79 | 0.80 | 0.74 | 0.72 |
|  | **R-square** | 0.51 | 0.58 | 0.66 | 0.63 | 0.64 | 0.55 | 0.52 |
|  | **RMSE** | 1.43 | 1.32 | 1.19 | 1.24 | 1.12 | 1.20 | 0.97 |
|  | **Skill Score (%)** | 50.91 | 58.11 | 65.78 | 63.05 | 64.04 | 54.69 | 51.86 |
|  | **Strike Rate – exact (%)** | 32 | 22 | 36 | 40 | 54 | 38 | 38 |
|  | **Strike Rate - ±1 (%)** | 68 | 70 | 76 | 76 | 78 | 78 | 92 |
|  | **AICc** | 181.94  210.32 | 174.05  210.32 | 163.91  210.32 | 167.77  210.32 | 157.66  201.46 | 164.54  196.20 | 143.03  169.84 |
|  | **AICc difference**  **(model-intercept only)** | 28.38 | 36.27 | 46.42 | 42.55 | 43.81 | 31.66 | 26.81 |
| **Tonga** | **Model Number** | 3 | 7 | 9 | 7 | 5 | 4 | 9 |
|  | **Correlation (r)** | 0.55 | 0.64 | 0.67 | 0.62 | 0.60 | 0.62 | 0.61 |
|  | **R-square** | 0.30 | 0.41 | 0.45 | 0.38 | 0.36 | 0.39 | 0.37 |
|  | **RMSE** | 1.34 | 1.24 | 1.19 | 1.27 | 1.27 | 1.20 | 0.98 |
|  | **Skill Score (%)** | 30.08 | 40.61 | 45.39 | 38.04 | 35.76 | 38.80 | 36.59 |
|  | **Strike Rate – exact (%)** | 30 | 24 | 36 | 34 | 28 | 22 | 38 |
|  | **Strike Rate - ±1 (%)** | 76 | 74 | 78 | 78 | 76 | 82 | 90 |
|  | **AICc** | 175.93  182.58 | 167.79  182.58 | 163.58  182.58 | 169.90  182.58 | 170.22  182.26 | 164.99  178.49 | 144.80  155.43 |
|  | **AICc difference**  **(model-intercept only)** | 6.66 | 14.79 | 19.00 | 12.68 | 12.04 | 13.50 | 10.63 |
| **Vanuatu** | **Model Number** | 10 | 6 | 2 | 1 | 9 | 9 | 4 |
|  | **Correlation (r)** | 0.65 | 0.71 | 0.69 | 0.70 | 0.73 | 0.73 | 0.59 |
|  | **R-square** | 0.43 | 0.51 | 0.48 | 0.50 | 0.53 | 0.54 | 0.35 |
|  | **RMSE** | 1.18 | 1.09 | 1.13 | 1.11 | 1.01 | 0.94 | 0.81 |
|  | **Skill Score (%)** | 42.48 | 50.92 | 47.79 | 49.63 | 52.70 | 53.70 | 35.02 |
|  | **Strike Rate – exact (%)** | 36 | 38 | 32 | 48 | 38 | 44 | 46 |
|  | **Strike Rate - ±1 (%)** | 80 | 90 | 82 | 82 | 90 | 90 | 92 |
|  | **AICc** | 162.98  182.62 | 155.09  182.62 | 158.21  182.62 | 156.41  182.62 | 147.24  178.34 | 140.40  172.03 | 125.12  143.44 |
|  | **AICc difference**  **(model-intercept only)** | 19.64 | 27.53 | 24.42 | 26.22 | 31.10 | 31.63 | 18.32 |


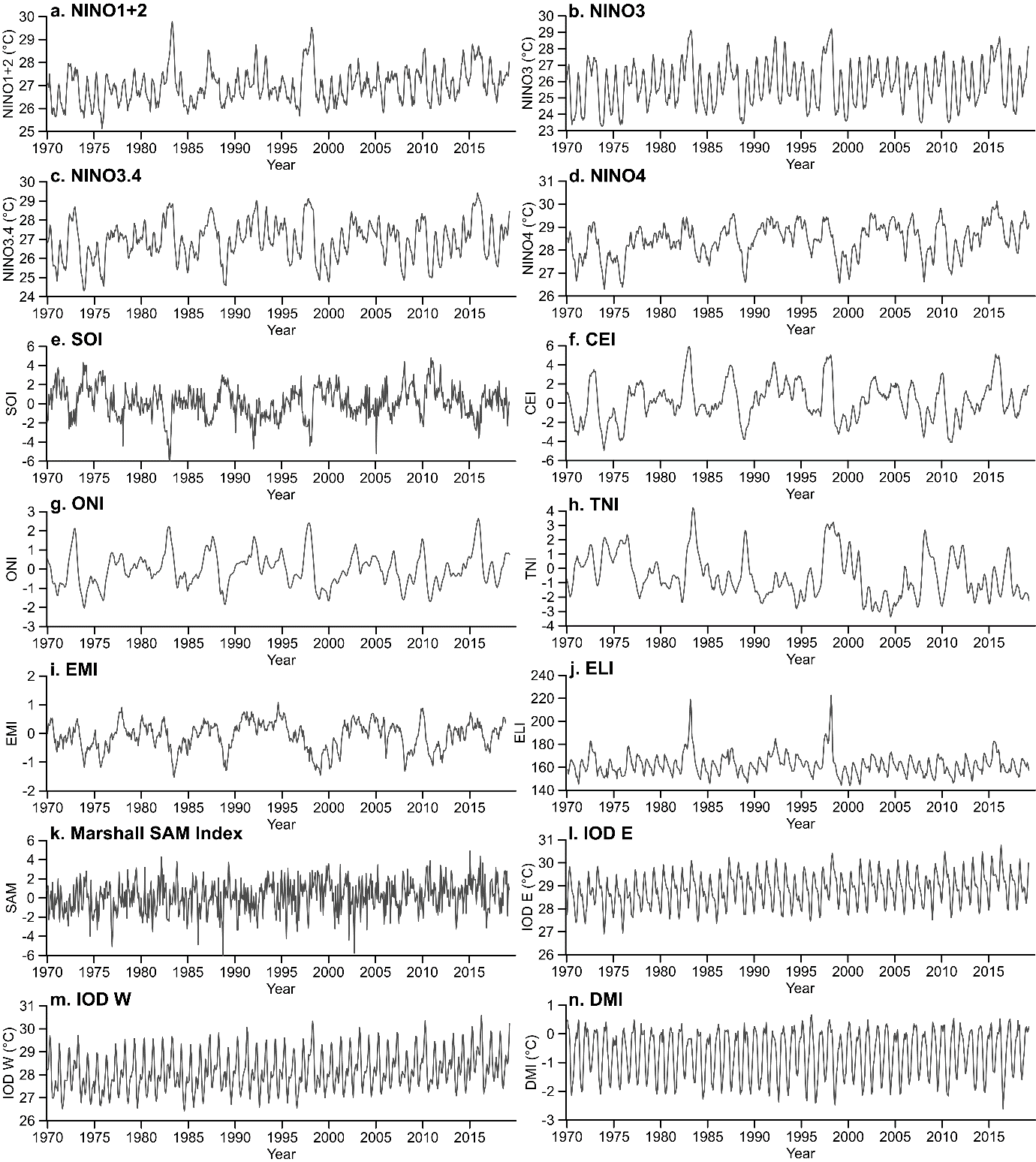


**Figure S1.** Time series of monthly indices between 1970-2019 for all indices considered in this analysis. Panels a-j are ENSO Indices. See Data and Model Development section for more details on each of these indices.
